# Qualitative Evaluation of Project P.A.T.H.S.: An Integration of Findings Based on Program Participants

**DOI:** 10.1100/2012/528483

**Published:** 2012-05-15

**Authors:** Daniel T. L. Shek, Rachel C. F. Sun

**Affiliations:** ^1^Department of Applied Social Sciences, The Hong Kong Polytechnic University, Hong Kong; ^2^Public Policy Research Institute, The Hong Kong Polytechnic University, Hong Kong; ^3^Department of Social Work, East China Normal University, Shanghai 200062, China; ^4^Kiang Wu Nursing College of Macau, Macau; ^5^Division of Adolescent Medicine, Department of Pediatrics, Kentucky Children's Hospital, University of Kentucky College of Medicine, Lexington, KY 40536, USA; ^6^Faculty of Education, The University of Hong Kong, Hong Kong

## Abstract

An integration of the qualitative evaluation findings collected in different cohorts of students who participated in Project P.A.T.H.S. (Positive Adolescent Training through Holistic Social Programmes) (*n* = 252 students in 29 focus groups) was carried out. With specific focus on how the informants described the program, results showed that the descriptions were mainly positive in nature, suggesting that the program was well received by the program participants. When the informants were invited to name three metaphors that could stand for the program, positive metaphors were commonly used. Beneficial effects of the program in different psychosocial domains were also voiced by the program participants. The qualitative findings integrated in this paper provide further support for the effectiveness of the Tier 1 Program of Project P.A.T.H.S. in promoting holistic development in Chinese adolescents in Hong Kong.

## 1. Introduction

 There are two contrasting research approaches in social sciences [1]. Having its root in positivism, the quantitative approach of research design has several characteristics. First, it relies on empirical methods with clear rules and procedures, deductive methods, and hypothesis testing. Second, value neutrality (i.e., suspension of judgment of the researchers) is strongly emphasized. Third, representativeness and generalization of the findings to explain social phenomena and predict outcomes are upheld. Fourth, quantification of the results is emphasized with the use of mathematical models, statistical analyses, and presentations. Fifth, validity, reliability, and objectivity are hallmarks of positivistic research [2, 3]. While the quantitative research approach has been the “mainstream” approach in the past decades, and its strengths are appreciated by disciplines, particularly those in the biomedical field, it has been criticized on its ontological and methodological assumptions. Ontologically, the assumption that the reality is “objective” and “out there” is questioned. For example, Patton [4] criticized the quantitative-experimental approach in terms of its oversimplification of the real world, the fact that it misses major factors of importance that are not easily quantified, and its failure to examine the holistic impact of a program. In addition, quantitative research is criticized as not being able to examine the essence of life of human beings. Finally, with its artificial nature, quantitative research is criticized as neglecting subjective experiences and interpreted meanings of the “actors.”

Because of the limitations of positivistic research, there is a growing emphasis on qualitative research in social sciences [5]. Qualitative research is defined as “an umbrella term for an array of attitudes toward and strategies for conducting inquiry that are aimed at discerning how human beings understand, experience, interpret, and produce the social world” ([6, page 893]). Unlike quantitative research that has a homogenous philosophical base, the qualitative approach includes a variety of philosophical positions and methodological approaches arising from different foundations. There are several attributes of qualitative research. First, a wide range of research methods (e.g., interviews, focus groups, observations, documentation) are commonly used. Second, the impossibility of value neutrality is acknowledged and usually addressed in a disciplined manner. Third, idiographic and uniqueness of individual cases rather than representativeness and generalization of the findings are emphasized. Fourth, there is weak reliance on “numbers,” while real-life data, such as narratives and lived experiences, are focused upon. Fifth, reliance of credibility, authenticity, and world views of the informants are hallmarks of qualitative research [7]. Of course, there are criticisms that qualitative research may lack methodological rigor and that it is a relatively “softer” form of research.

These two main approaches of research are also seen in the field of evaluation. In the biomedical fields, the experimental and quantitative evaluation method is commonly regarded as the “gold” standard in assessing the outcomes of a program. In contrast, in social service settings, such as the fields of social work and education, the nonexperimental and qualitative evaluation method is commonly used to understand the process of implementing the program and the lived experiences of the program participants. As pointed out by Patton [8], there is a general consensus in the field of evaluation that a sole reliance of either a quantitative or qualitative method may not be adequate in understanding the effect of a program.

Project P.A.T.H.S. (Positive Adolescent Training through Holistic Social Programmes) is a youth enhancement program that attempts to promote holistic youth development in Hong Kong [9]. There are two tiers of programs (Tier 1 and Tier 2 Program) in this project. The Tier 1 Program is a universal positive youth development program based on 15 positive youth development constructs [10] in which students in Secondary 1 to 3 take part. To date, many evaluation studies have been conducted in order to examine the effectiveness of the program. For example, adopting a randomized group trial based on experimental design, research findings showed that participants in the experimental group had better development, but less problem behavior, than did the control group participants [11–13]. Similarly, subjective outcome evaluation utilizing quantitative rating scales had been used in order to understand the perceptions of the program participants and implementers [14–16]. The findings generally revealed that program participants and implementers had positive perceptions of the program and implementers, and they regarded the program as beneficial to the development of the program participants. While the above evaluation findings based on quantitative methods are valuable, it is equally important to understand the views of the program participants via a qualitative approach. As such, qualitative methods, such as focus groups, are valuable tools for understanding the views of the program participants.

In a pioneering focus group study conducted by Shek et al. [17], five focus groups based on 43 students recruited from four schools were conducted in order to generate qualitative data to evaluate the program. With specific focus on how the informants described the program, results showed that the descriptors used were mainly positive in nature. When the informants were invited to name three metaphors that could stand for the program, the related metaphors were basically positive in nature. Finally, the program participants perceived many beneficial effects of the program in different psychosocial domains. Similarly, Shek and Lee [18] conducted 10 focus groups comprising 88 students recruited from 10 schools in order to understand the perceptions of students participating in the Tier 1 Program of P.A.T.H.S. Project. Results showed that a majority of the participants described the program positively and they perceived beneficial effects of the program in several aspects of adolescent lives. Similar findings were shown based on other cohorts of students [19].

As a research methodology, focus groups have emerged as a popular tool for generating qualitative data and are used across a wide variety of disciplines and applied research areas [20]. Since the 1980s, there has been a growing use of focus groups, particularly in health research [21]. In his review of online databases, Morgan [22] reported that focus groups appeared in 100 academic journal articles per year throughout the decade, and he also observed that focus groups were always used in conjunction with other research methods. According to Morgan and Spanish [23], “as a qualitative method for gathering data, focus groups bring together several participants to discuss a topic of mutual interest to themselves and the researcher” (p. 253). Similarly, Basch [24] defined focus groups as “a qualitative research technique used to obtain data about feelings and opinions of small group of participants about a given problem, experience, service or other phenomenon” (p. 414).

There are several advantages of focus groups [25]. Primarily, the dynamic group process and interaction of group members can generate useful information for the researchers [26]. Likewise, Twinn [27] stated that the synergism created by the interaction of group members is important to the generation of ideas that could be difficult to obtain through individual interviews. Focus groups are also advantageous in handling complicated topics in a relatively short period of time, particularly when the objective of focus groups is to collect nonconsensual data [28], and they can gather data at a lower cost than any other qualitative research method [29].

Interestingly, in spite of its current popularity in different fields of social sciences, little has been documented about the use of the focus group methodology in program evaluation. Ansay et al. [30] highlighted that “although focus groups continue to gain popularity in marketing and social science research, their use in program evaluation has been limited” (p. 310). To date, there is sparse scientific evidence on the use of focus groups within the Chinese adolescent population in program evaluation, despite the fact that focus groups are considered to be an effective qualitative data technique that is readily understood by program funders [31]. This paper therefore attempts to fill this gap in the literature with specific focus on P.A.T.H.S. Project. Based on several cohorts of data collected via focus groups in the project, the present study attempts to integrate the findings in the existing cohorts and produce an integrated picture on the views of the program participants.

Although the focus group as a qualitative method is widely used, it has been criticized as lacking rigor [32]. Therefore, some guidelines for enhancing the quality of qualitative research should be maintained. In their review of the common problems intrinsic to qualitative evaluation studies in the social work literature, Shek et al. [33] suggested that 12 principles should be maintained in a qualitative evaluation study. These include the following: explicit statement of the philosophical base of the study (Principle 1); justifications for the number and nature of the participants of the study (Principle 2); detailed description of the data collection procedures (Principle 3); discussion of the biases and preoccupations of the researchers (Principle 4); description of the steps taken to guard against biases or arguments that biases should and/or could not be eliminated (Principle 5); inclusion of measures of reliability, such as inter- and intrarater reliability (Principle 6); inclusion of measures of triangulation in terms of researchers and data types (Principle 7); inclusion of peer and member checking procedures (Principle 8); consciousness of the importance and development of audit trails (Principle 9); consideration of alternative explanations for the observed findings (Principle 10); inclusion of explanations for negative evidence (Principle 11); clear statement of the limitations of the study (Principle 12). It was argued that the above principles should be upheld as far as possible in focus group studies. In the focus group studies integrated in this paper, these principles were adopted as far as possible.

The purpose of this paper is to present an integrated picture of the qualitative findings collected in a series of focus group studies with students participating in the Tier 1 Program of P.A.T.H.S. Project. In each focus group study, a general qualitative research approach [34] was adopted, where general strategies of qualitative research were employed (e.g., collection of qualitative data, respecting the views of the informants, data analysis without preset coding scheme), but a specific qualitative approach was not adhered to. The exposition of the nature of this qualitative study is consistent with the view of Shek et al. [33] that there should be an explicit statement of the philosophical base of the study (Principle 1).

## 2. Methods

### 2.1. Participants and Procedures

From 2005 to 2009, in the Experimental and Full Implementation Phases, the total number of schools that participated in Project P.A.T.H.S. was 244, with 669 schools in the Secondary 1 level, 443 in the Secondary 2 level, and 215 in the Secondary 3 level. Among them 46.27% of the respondent schools adopted the full program (i.e., 20 h program involving 40 units), whereas 53.73% of the respondent schools adopted the core program (i.e., 10 h program involving 20 units).

A total of 28 schools were randomly selected for the study of student focus group evaluation (14 schools for the Secondary 1 program, 10 for the Secondary 2 program, and four for the Secondary 3 program), in which 23 schools joined the full program (20 h) and five schools joined the core program (10 h) of the Tier 1 Program of P.A.T.H.S. Project. Among the schools that joined this study, 67.9% (*n* = 19) incorporated the Tier 1 Program into the formal curriculum (e.g., Liberal Studies, Life Education, and Religious Studies) and 32.1% (*n* = 9) used the class teacher's period or other modes to implement the program. For the consenting schools, the respective workers randomly selected students to join the focus groups. In all, 252 students joined 29 focus groups of approximately 1 h each, with the number of informants in each focus group ranging from 3 to 12 students. The characteristics of the schools that joined this qualitative evaluation study can be seen in Table 1.

Because data collection and analyses in qualitative research are very labor intensive, it is the usual practice that small samples are used. In the present context, the number of focus groups and student participants could be regarded as respectable. In addition, the strategy of randomly selecting informants and schools that joined the project could help to enhance the generalizability of the findings. These arguments can satisfy Principle 2 (i.e., justifications for the number and nature of the participants of the study) proposed by Shek et al. [33].

### 2.2. Instruments

An interview guide was used for conducting focus group interviews with students (Table 2). In the focus group studies under review, qualitative analyses were analyzed mainly in three areas: (1) descriptors that were used by the informants to describe the program, (2) metaphors (i.e., incidents, objects, or feelings) that were used by the informants to stand for the program, and (3) informants' perceptions of the benefits of the program to themselves. To enhance credibility of the findings, the data were analyzed by two trained research assistants and crosschecked by another trained research assistant. Furthermore, to enhance the reliability of the coding on the positivity nature of the raw codes, both intra- and interrater reliability were carried out. Results in the focus group studies reviewed in this study showed that the intra- and interrater reliability were on the high side [17–19]. The raw data and categorized data were kept in a systematic filing system in order to ensure that the findings were auditable.

## 3. Results

There were 390 raw descriptors used by the informants to describe the program, and they could be further categorized into 78 categories (see Table 3). Among these descriptors, 234 (60%) were coded as positive descriptors, whereas 120 (30.8%) could be classified as negative descriptors. In order to examine the reliability of the coding, two research assistants who did the coding of raw data recoded 20 randomly selected raw descriptors at the end of the scoring process, and the average intrarater agreement percentage calculated on the positivity of the coding from these descriptors was 96.3% (range 90–100%). Finally, these 20 randomly selected descriptors were coded by another two research staff members who did not know the original codes given, and the average inter-rater agreement percentage calculated on the positivity of the coding was 94.4% (range 90–100%).

For the metaphors that were used by the informants that could stand for the program, there were 188 raw objects involving 242 related attributes (Table 4). Results showed that 109 metaphors (58%) and 158 attributes (65.3%) could be classified as positive in nature, and 43 metaphors (22.9%) and 41 related attributes (16.9%) were regarded as neutral responses. Reliability tests showed that the average intrarater agreement percentage calculated on the positivity of the coding from these metaphors was 96.3% (range 92.5–100%), whereas the average inter-rater agreement percentage calculated on the positivity of the coding was 88.8% (range 85–95%).

The perceived benefits of the program to the program participants are shown in Table 5. There were 754 meaningful responses decoded from the raw data categorized into several levels, which are benefits at societal, familial, interpersonal, and personal levels and general benefits. Most of the perceived benefits to program participants fell on the personal level (*n* = 305), followed by benefits on the interpersonal level (*n* = 152). The findings showed that 597 responses (79.2%) were coded as positive responses and 35 responses (4.6%) were counted as neutral responses. In order to examine the reliability of coding, the research assistants recoded 20 randomly selected responses at the end of the scoring process. The average intrarater agreement percentage calculated from these responses was 98.1% (range 95–100%). The raw benefit categories were coded again by another two research staff members who did not know the original codes given. The average inter-rater agreement percentage calculated from these responses was 95% (range 90–97.5%).

## 4. Discussion

The purpose of this study was to evaluate the Tier 1 Program of Project P.A.T.H.S. using findings based on focus groups involving program participants in the Experimental and Full Implementation Phases (2005–2009) of the project. There are several characteristics of this study. First, a large sample of participants (*n* = 252 students in 28 secondary schools) were involved. Second, different datasets collected at different points of time were analyzed. Third, views of students in different grades were collected. Fourth, this is the first known scientific study of focus group evaluation of a positive youth development program based on different cohorts in China. Finally, this is also the first focus group evaluation study based on such a large sample of participants in the global context.

Based on the integrative analyses, two salient observations can be highlighted from the findings collected from different cohorts of students. First, the program was generally perceived as positive from the perspective of the program participants (Table 3), although some students perceived the program to be negative, which was not the dominant view. The program participants generally used positive descriptors and metaphors to describe the program (Table 4). Nevertheless, some negative responses were recorded, although those were not the dominant responses.

Second, results in Table 5 show that the program had beneficial effects on the participants, with roughly 80% of the responses coded as positive. Generally speaking, benefits in both the personal and interpersonal levels were observed. The above observations are generally consistent with the objective outcome evaluation findings [11–13] that the students changed in the positive direction in various developmental domains. With reference to the principle of triangulation, the present study and the previous findings suggest that based on both quantitative and qualitative evaluation findings, evidence on the positive effects of the Tier 1 Program on holistic youth development among the program participants is present.

As suggested by Shek et al. [33], it is imperative to consider alternative explanations in the interpretations of qualitative evaluation findings (Principle 10). There are several plausible alternative justifications for the findings based on the focus group methods. The first alternative explanation is demand characteristics. However, this explanation is not likely because the participants were encouraged to express their views freely and negative voices were, in fact, heard. In addition, since the teachers were not present, there was no need for the students to respond in a socially desirable manner. Another explanation is that the findings were due to selection bias. However, this argument is not strong because the schools and students were randomly selected. The third explanation is that the positive findings were due to ideological biases (e.g., self-fulfilling prophecies) of the researchers. However, because several safeguards were used to reduce biases in the data collection and analysis processes, this possibility is not high. Finally, it may be argued that the perceived benefits were due to other youth enhancement programs. Nonetheless, this argument can be partially dismissed because none of the schools in the present study joined the major youth enhancement programs in Hong Kong, including the Adolescent Health Project and the Understanding the Adolescent Project. Most importantly, participants in the focus group interviews were specifically asked about the program effects of Project P.A.T.H.S. only.

There are several contributions of the present study. First, in view of the lack of positive youth development programs and related evaluation findings in the Chinese contexts, the present study is pioneering. Besides showing that Project P.A.T.H.S. is effective, it also demonstrates how focus group evaluation based on a large sample can be carried out. Second, the present integrative study demonstrates how the principle of qualitative evaluation studies proposed by Shek et al. [33] could be applied in focus group studies. Finally, the findings demonstrate the utility of using “descriptors” and “metaphors” in generating qualitative data. Actually, a review of the literature shows that there is an increasing effort to conduct qualitative evaluation studies. Bowey and McGlaughlin [35] studied the views of 11 young persons with an objective to improve attitudes to crime and the police, to reduce exclusion, and to develop self-esteem in at-risk young people. De Anda [36] collected qualitative data to evaluate the first year of a mentor program for at-risk high school youth in a low-income urban setting. Nicholas et al. [37] collected qualitative data from 24 adolescents with chronic kidney disease to evaluate an online social support network. The present study further illustrates the utility of collecting qualitative data in evaluation contexts.

On the other hand, there are several limitations of the study that should be addressed in qualitative research (Principle 12). Primarily, several general limitations involved in focus groups are worth noting. First, focus groups provide descriptions about the perceptions of the program, and they are not useful for testing hypotheses in the traditional experimental design. Second, although group interaction is generally seen as an advantage of focus groups, there is always the possibility that intimidation within the group setting may inhibit interaction. Further, caution must also be exercised because the quality of the findings is tied to the skills of the moderator. Regarding the second and third limitations, the use of experienced moderators in this study could minimize the problems. In addition, the inclusion of other qualitative evaluation strategies, such as in-depth individual interviews, would be helpful to further understand the subjective experiences of the program participants. Despite these limitations, the present study provides pioneering qualitative evaluation findings supporting the positive nature of Project P.A.T.H.S. and its effectiveness in promoting holistic youth development among Chinese adolescents in Hong Kong.

## Figures and Tables

**Table 1:** Description of data characteristics from 2005 to 2009.

	2005/06 (EIP-S1)	2006/07 (FIP-S1)	2007/08 (FIP-S2)	2007/08 (EIP-S3)
Total schools that joined P.A.T.H.S.	52	207	196	48
(i) 10 h program	23	95	113	29
(ii) 20 h program	29	112	83	19
Total schools that joined this study	4	10	10	4
(i) 10 h program	1	2	2	0
(ii) 20 h program	3	8	8	4
(a) No. of schools incorporated into formal curriculum	3	4	8	4
(b) No. of schools incorporated into class teacher's period and other time slots	1	6	2	0
Average no. (range) of classes per school	5 (5)	4.9 (3–6)	4.9 (3–6)	4.8 (4–6)
No. of student focus groups	5^a^	10	10	4
Total student respondents	43	88	92	29
Average no. (range) of respondents per group	8.6 (3–10)	8.8 (4–12)	9.2 (4–11)	7.3 (3–10)

Note: EIP = experimental implementation phase, FIP = full implementation phase, S1: Secondary 1 level; S2: Secondary 2 level; S3: Secondary 3 level.

^a^Eleven students in a school were divided into two focus groups.

**Table 2:** Interview guide for the student focus group.

	(A) Process Evaluation:
	(1) Comments on the Program Content
	(i) Were there any activities that most effectively aroused your interest to participate in them?
	(ii) Regarding the program, what are the things you like? What are the things you dislike?
	(iii) What are your views on the different units and content of the program?
	(iv) Which units do you like the most? Why?
	(2) Comments on the Program Implementation
	(i) What are your thoughts on the degree or extent of participation of the entire class (all the students)?
	(ii) How do you feel about the atmosphere and discipline of the class when the program was implemented?
	(iii) What are the responses of the participating students regarding the program?
	(3) Comments on the Instructors
	(i) What are your views on the instructors who conducted the program?
	(ii) Regarding the interactions between the instructors and students, what are your thoughts and feelings?
	(B) Product Evaluation:
	(1) Evaluation of the General Effectiveness of the Program
	(i) Do you feel that the program is beneficial to the development of adolescents?
	(ii) Do you think that the program has helped your development?
	(iii) After participating in the program, do you have any changes? If yes, please specify. (free elicitation)
	(iv) What have you gained in this program? (free elicitation)
	(v) If you feel that you have changed, what do you think are the factors that have promoted such changes?
	(vi) If you have not noticed any changes in yourself, what do you think are the reasons?
	(2) Evaluation of the Specific Effectiveness of the Program
	(i) Do you think that your participation in the program has affected your schoolwork and grades? Please elaborate your answers.
	(ii) Do you think the program can promote your self-confidence or ability to face the future?
	(iii) Do you think the program can enhance your abilities in different areas in your life?
	Optional Questions
	(i) Do you think the program can promote your spiritual life?
	(ii) Do you think the program can promote your bonding with family, teachers, and friends?
	(iii) Do you think the program can cultivate your compassion and caring for others?
	(iv) Do you think the program can promote your participation and caring for society?
	(v) Do you think the program can promote your sense of responsibility to society, family, teachers, and peers?
	(3) Other Comments
	(i) If you are invited to use three descriptors to describe the program, what three words will you use?
	(ii) If you are invited to use one incident, object, or feeling (e.g., indigestion, enjoyment, etc.) to describe the program, what metaphors will you use to stand for the program?

**Table 3:** Categorization of the descriptors used by the students to describe the program.

Descriptors	2005/06 (EIP-S1)	2006/07 (FIP-S1)	2007/08 (FIP-S2)	2007/08 (EIP-S3)	Total (percentage of total responses)
Positive responses					
Fun, amusing	10	5	12	4	31
Interesting	7	8			15
Good, excellent	6	6	4	2	18
Lively, exciting, not dull	5	3	8	3	19
Meaningful	4	2	2		8
Novel	3	4			7
Relaxed	3	6	10		19
Comfortable, enjoyable, confident	3	4	1		8
Happy	3	12	12	4	31
Rich content	2	2	4	1	9
Comprehensible	2				2
Applicable, close to real life	2	4			6
Useful	1		1		2
Professional	1				1
Better than other lessons	1				1
Efficient	1				1
Smooth		1			1
Time field (because of enjoyment)		1			1
Helpful/constructive		2			2
Active		1			1
Looking forward to attend the program		1			1
Reflecting		2			2
Enlightening		2			2
In depth		1			1
Involvement		1			1
Direct		1			1
Ability		1			1
Good luck		1			1
Surprising			3		3
Learn a lot			1		1
Meet the needs of students			2		2
Beneficial			2	2	4
Pride			2		2
Fruitful			4		4
Energetic			1		1
Perfect			1		1
Motivating			1		1
Like a teacher			1		1
Attractive			1	1	2
Outstanding			1		1
Satisfied			3	2	5
Serious			1	1	2
Delicious				1	1
Practical				7	7
Unique				1	1
Good atmosphere				2	2

Subtotal (percentage of total responses in each academic year)	54 (76.1)	71 (64)	78 (54.2)	31 (48.4)	234 (60)

Negative responses					
Boring	10	10	11	6	37
Senseless	3	9	13	4	29
Repetitive	1				1
Killing time	1	1			2
Helpless		3			3
Horrible		2			2
Without novelty		1			1
Meaningless		1	1		2
Disappointing		1			1
Inflexible		1			1
Passive		2			2
Chaotic		1		1	2
Monotonous			3		3
Empty			1	1	2
Troublesome			4		4
Waste of time			8	1	9
Not interactive			1		1
Too relaxing			3		3
Annoying			2	1	3
Useless			2	4	6
Unattractive				3	3
Unhappy				1	1
Naïve				2	2

Subtotal (% of total responses in each academic year)	15 (21.1)	32 (28.8)	49 (34)	24 (37.5)	120 (30.8)

Neutral responses					
Fair	1		7	1	9
To be improved			2	4	6

Subtotal (% of total responses in each academic year)	1 (1.4)	0	9 (6.2)	5 (7.8)	15 (3.9)

Undecided					
Low cost	1	1			2
Unlike a class			1		1
Have no feelings on the program			3		3

Subtotal (% of total responses in each academic year)	1 (1.4)	1 (0.9)	4 (2.8)	0	6 (1.5)

Other comments					
Positive		3			3
Negative		1			1
Neutral		2			2
Undecided		1	4	4	9

Subtotal (% of total responses in each academic year)	0	7 (6.3)	4 (2.8)	4 (6.3)	15 (3.9)

Total count	71 (100)	111 (100)	144 (100)	64 (100)	390 (100)

**Table 4:** Metaphors used by participants to describe the program.

Nature of response	No. of responses towards the nature of metaphor				
	2005/06 (EIP-S1)	2006/07 (FIP-S1)	2007/08 (FIP-S2)	2007/08 (EIP-S3)	Total (%)

Positive items (%)	27	36	32	14	109
(e.g., mirror, stair, rainbow, sunshine after rain)	(77.1)	(51.4)	(56.1)	(53.8)	(58)
Negative items (%)	5	9	17	5	36
(e.g., beat each other, invisible pen, disappointment, talking tactics on paper)	(14.3)	(12.9)	(14.0)	(19.2)	(19.1)
Neutral items (%)	3	25	8	7	43
(e.g., train, watching movie, parenting, medicine)	(8.6)	(35.7)	(29.8)	(26.9)	(22.9)
Undecided items (%)	0	0	0	0	0
(e.g., zip file, white paper)					

Total count (%)	35 (100)	70 (100)	57 (100)	26 (100)	188 (100)

	No. of codes derived from the metaphor				

Positive items (%)	33	58	43	24	158
(e.g., mirror, stair, rainbow, sunshine after rain)	(82.5)	(66.7)	(57.3)	(60)	(65.3)
Negative items (%)	4	8	24	5	41
(e.g., beat each other, invisible pen, disappointment, talking tactics on paper)	(10)	(9.2)	(32)	(12.5)	(16.9)
Neutral items (%)	3	21	7	10	41
(e.g., train, watching movie, parenting, medicine)	(7.5)	(24.1)	(9.3)	(25)	(16.9)
Undecided items (%)	0	0	1	1	2
(e.g., zip file, white paper)			(1.3)	(2.5)	(0.8)

Total count (%)	40 (100)	87 (100)	75 (100)	40 (100)	242 (100)

**Table 5:** Categorization of responses on the perceived benefits of and things learned in the program.

Area of competence	Subcategory	Benefits	2005/06 (EIP-S1)	2006/07 (FIP-S1)	2007/08 (FIP-S2)	2007/08 (EIP-S3)	Total
Societal level	Social responsibility and affairs	Learned voluntary work	1	5			6
		Enhanced understanding of mother country	2	4			6
		Enhanced sense of contribution to society	0	2			2
		Increased awareness of different social issues			2		2
		*Subtotal*	3	11	2	0	16

Familial level	Family relationships	Improved communication and relationship with family	7	11	1	6	25
		*Subtotal*	7	11	1	6	25

Interpersonal level	General interpersonal competence	Enhanced teacher-student relationship and understanding	6	7	4	2	19
		Learned teamwork	1	7			8
		Enhanced mutual support		7			7
		Improved relationship with peers/made more friends	17	5	5		27
		Able to differentiate between good and bad friends	1	4			5
		Strengthened connection with healthy adults			1		1
		*Total in subcategory*	25	30	10	2	67
	Specific interpersonal competence	Enhanced interpersonal skills			11	3	14
		Improved communication skills and interpersonal relationship	10	15	3		28
		Used learned materials to help or teach others	2				2
		Learned how to handle conflicts/avoid conflicts	3	3	1		7
		Learned how to treat people and deal with issues	2	1			3
		Learned to share and express oneself	4	3			7
		Promoted mutual understanding among peers		6			6
		Leadership		2			2
		Learned to respect others	1		1		2
		Reduced bullying behavior	2				2
		Became a good listener			2		2
		Learned to take care of others			1	1	2
		Better understanding of others			5		5
		Learned to accept others' opinions			2		2
		Learned to make apology			1		1
		*Total in subcategory*	24	30	27	4	85
		*Subtotal*	49	60	37	6	152

Personal level	Behavioral competence	Acquired refusal skills	3	1	1	1	6
		Promoted presentation skills		8	6		14
		Took initiative		6	3		9
		Put more effort on studies			1		1
		Learned to work seriously			1		1
		Increased willingness in trying new things			1		1
		*Total in subcategory *	3	15	13	1	32
	Cognitive competence	Enhanced problem-solving skills	13	5	4		22
		Learned critical thinking	1	6	10	3	20
		Open minded			1		1
		Able to distinguish between right or wrong			2		2
		*Total in subcategory*	14	11	17	3	45
	Emotional competence	Enhanced stress management/relaxation	3	5		1	9
		Enhanced ability in handling emotions	9	8			17
		Enhanced anger management, became less impulsive	4				4
		Developed good temper			2		2
		Enhanced emotional management			11	1	12
		*Total in subcategory*	16	13	13	2	44
	Moral competence and virtues	Learned to do appropriate things at the right place/right time	3	8			11
		Enhanced empathy	1	4	2		7
		Increased awareness of public morals	1	4			5
		Enhanced sense of equality		2			2
		Understood personal responsibility		1			1
		Able to correct bad habits			1		1
		*Total in subcategory*	5	19	3	0	27
	Beliefs in the future	Facilitated goal setting and realization of goals	1	5	4	5	15
		Helpful for future career	2				2
		Prepared for the future			2	2	4
		*Total in subcategory*	3	5	6	7	21
	Positive self-image	Enhanced self-understanding	4	13	7		24
		Promoted self-enrichment		3			3
		Enhanced personal growth		1	4	5	10
		Enhanced self-confidence	6	18	10	2	36
		Enhanced self-efficacy	1	1			2
		Enhanced self-discipline	1				1
		Be more active		2			2
		Enhanced self-determination		2	1	2	5
		Identified one's strengths			1		1
		Gained wisdom			2		2
		Had little personal changes			1		1
		*Total in subcategory*	12	40	26	9	87
	Cherishing life	Cherishing life, people, and things		5		1	6
		Enhanced self-reflection	1	2	4		7
		Helpful in understanding purpose of life	1	4			5
		*Total in subcategory*	2	11	4	1	18
	Resilience	General resilience				1	1
		Learned positive thinking	1	3	4	3	11
		Be more persistent when facing difficulties		1			1
		Learned ways to face adversity			6		6
		Be optimistic			1	1	2
		*Total in subcategory*	1	4	11	5	21
	General gains	Had positive personality change	4				4
		Learned from failures	1				1
		Enhanced feeling of being supported	1				1
		Enhanced motivation for learning				1	1
		Better academic achievement				3	3
		*Total in subcategory *	6	0	0	4	10
		*Subtotal*	62	118	93	32	305

General benefits	Positive comments	Learned many things	2	9			11
		Learned practical things		2			2
		Helpful/very helpful		5			5
		Met students' needs			4		4
		Provided opportunities for students to share			1		1
		Better than normal lessons			1		1
		Benefit to study		8			8
		Enhanced bonding to school	1				1
		Enhanced concentration in class			2		2
		Others			19	3	22
		*Total in subcategory*	3	24	27	3	57
	Negative comments	Could not learn anything	2	9			11
		Unhelpful	2	19			21
		Not much change		6			6
		Not beneficial to academic studies		2	1		3
		Negative change		2			2
		Others			4	7	11
		*Total in subcategory*	4	38	5	7	54
	Neutral comments	Some of the content useful, but some useless		6			6
		Not very helpful		2			2
		Not much change	1	2			3
		Not much helpful to study		1			1
		Not much change in teacher-student relationship and understanding		3			3
		Learned practical things		1			1
		Others				1	1
		*Total in subcategory*	1	15	0	1	17
	Undecided	The change was doubtful		2			
		*Total in subcategory*	0	2	0	0	2
		*Subtotal*	8	79	32	11	130

Others	Other comments	Positive comments		3	32	7	42
		Negative comments		2	29	22	53
		Neutral comments		2	1	15	18
		Undecided		3	7	3	13
		*Subtotal*	0	10	69	47	126

Total count			129	289	234	102	754

## References

[1] Leung JTY, Shek DTL (2011). Quantitative and qualitative approaches in the study of poverty and adolescent development: separation or integration?. *International Journal of Adolescent Medicine and Health*.

[2] Manicas P, Outhwaite W, Turner SP (2007). The social sciences since World War II: the rise and fall of Scientism. *The SAGE Handbook of Social Science Methodology*.

[3] Pelie C (1988). Research paradigms in social work: from stalemate to creative synthesis. *Social Service Review*.

[4] Patton MQ (1990). *Qualitative Evaluation and Research Methods*.

[5] Denzin NK, Lincoln YS (2005). *The Sage Handbook of Qualitative Research*.

[6] Lewis-Beck MS, Bryman A, Liao TF (2004). *The Sage Encyclopedia of Social Science Research Methods*.

[7] Guba EG, Lincoln YL, Denzin NK, Lincoln YS (1994). Competing paradigms in qualitative research. *Handbook of Qualitative Research*.

[8] Patton MQ (2008). *Utilization-Focused Evaluation*.

[9] Shek DTL, Sun RCF (2009). Development, implementation and evaluation of a holistic positive youth development program: project P.A.T.H.S. in Hong Kong. *International Journal on Disability and Human Development*.

[10] Catalano RF, Berglund ML, Ryan JAM, Lonczak HS, Hawkins JD http://aspe.hhs.gov/hsp/PositiveYouthDev99/.

[11] Shek DTL, Sun RCF (2010). Effectiveness of the tier 1 program of project P.A.T.H.S.: findings based on three years of program implementation. *TheScientificWorldJournal*.

[12] Shek DTL, Ma CMS (2011). Longitudinal data analyses using linear mixed models in SPSS: concepts, procedures and illustrations. *TheScientificWorldJournal*.

[13] Shek DTL, Yu L (2011). Prevention of adolescent problem behavior: longitudinal impact of the Project P.A.T.H.S. in Hong Kong. *TheScientificWorldJournal*.

[14] Ma HK, Shek DTL (2010). Subjective outcome evaluation of a positive youth development program in Hong Kong: profiles and correlates. *TheScientificWorldJournal*.

[15] Shek DTL, Sun RCF (2008). Evaluation of Project P.A.T.H.S. (Secondary 1 Program) by the program participants: findings based on the Full Implementation Phase. *Adolescence*.

[16] Shek DTL, Sun RCF (2010). Secondary data analyses of subjective outcome evaluation findings of the project P.A.T.H.S. in Hong Kong. *TheScientificWorldJournal*.

[17] Shek DTL, Lee TY, Siu AMH, Lam CM (2006). Qualitative evaluation of the project P.A.T.H.S. based on the perceptions of the program participants. *TheScientificWorldJournal*.

[18] Shek DTL, Lee TY (2008). Qualitative evaluation of the Project P.A.T.H.S.: findings based on focus groups with student participants. *International Journal of Adolescent Medicine and Health*.

[19] Shek DTL, Ng CSM, Tsui PF (2010). Qualitative evaluation of the Project P.A.T.H.S.: findings based on focus groups. *International Journal on Disability and Human Development*.

[20] Morgan DL (1996). Focus groups. *Annual Review of Sociology*.

[21] Kreuger RA (1994). *Focus Groups: A Practical Guide for Applied Research*.

[22] Morgan DL (1997). *Focus Groups as Qualitative Research*.

[23] Morgan DL, Spanish MT (1984). Focus groups: a new tool for qualitative research. *Qualitative Sociology*.

[24] Basch CE (1987). Focus group interview: an underutilized research technique for improving theory and practice in health education. *Health Education Quarterly*.

[25] Morgan DL, Krueger RA (1998). *The Focus Group Kit*.

[26] Asbury JE (1995). Overview of focus group research. *Qualitative Health Research*.

[27] Twinn S (2000). The analysis of focus group data: a challenge to the rigour of qualitative research. *Nursing Times Research*.

[28] White GE, Thomson AN (1995). Anonymized focus groups as a research tool for health professionals. *Qualitative Health Research*.

[29] Morgan DL (1993). *Successful Focus Groups: Advancing the State of the Art*.

[30] Ansay SJ, Perkins DF, Nelson CJ (2004). Interpreting outcomes: using focus groups in evaluation research. *Family Relations*.

[31] Nabors LA, Ramos V, Weist MD (2001). Use of focus groups as a tool for evaluating programs for children and families. *Journal of Educational and Psychological Consultation*.

[32] Webb C, Kevern J (2001). Focus groups as a research method: a critique of some aspects of their use in nursing research. *Journal of Advanced Nursing*.

[33] Shek DTL, Tang VMY, Han XY (2005). Quality of qualitative evaluation studies in the social work literature: evidence that constitutes a wakeup call. *Research on Social Work Practice*.

[34] Miles MB, Huberman AM (1994). *Qualitative Data Analysis*.

[35] Bowey L, McGlaughlin A (2006). The youth crime reduction video project: an evaluation of a pilot intervention targeting young people at risk of crime and school exclusion. *The Howard Journal of Criminal Justice*.

[36] De Anda D (2001). A qualitative evaluation of a mentor program for at-risk youth: the participants’ perspective. *Child and Adolescent Social Work Journal*.

[37] Nicholas DB, Picone G, Vigneux A (2009). Evaluation of an online peer support network for adolescents with chronic kidney disease. *Journal of Technology in Human Services*.

